# Nanographene‐Based Heterojunctions for High‐Performance Organic Phototransistor Memory Devices

**DOI:** 10.1002/advs.202300057

**Published:** 2023-03-30

**Authors:** Shaoling Bai, Lin Yang, Katherina Haase, Jakob Wolansky, Zongbao Zhang, Hsin Tseng, Felix Talnack, Joshua Kress, Jonathan Perez Andrade, Johannes Benduhn, Ji Ma, Xinliang Feng, Mike Hambsch, Stefan C. B. Mannsfeld

**Affiliations:** ^1^ Center for Advancing Electronics Dresden (cfaed) Technische Universität Dresden Helmholtzstraße 18 01062 Dresden Germany; ^2^ Faculty of Electrical and Computer Engineering Technische Universität Dresden Helmholtzstraße 18 01062 Dresden Germany; ^3^ Faculty of Chemistry and Food Chemistry Technische Universität Dresden Helmholtzstraße 18 01062 Dresden Germany; ^4^ Dresden Integrated Center for Applied Physics and Photonic Materials (IAPP) and Institute for Applied Physics Technische Universität Dresden Nöthnitzer Str. 61 01187 Dresden Germany; ^5^ Leibniz Institute for Solid State and Materials Research Helmholtzstraße 20 01069 Dresden Germany; ^6^ Max Planck Institute of Microstructure Physics Weinberg 2 06120 Halle Germany

**Keywords:** memory, nanographene, organic phototransistors, photosensitivity

## Abstract

Organic phototransistors can enable many important applications such as nonvolatile memory, artificial synapses, and photodetectors in next‐generation optical communication and wearable electronics. However, it is still a challenge to achieve a big memory window (threshold voltage response ∆*V*_th_) for phototransistors. Here, a nanographene‐based heterojunction phototransistor memory with large ∆*V*_th_ responses is reported. Exposure to low intensity light (25.7 µW cm^−2^) for 1 s yields a memory window of 35 V, and the threshold voltage shift is found to be larger than 140 V under continuous light illumination. The device exhibits both good photosensitivity (3.6 × 10^5^) and memory properties including long retention time (>1.5 × 10^5^ s), large hysteresis (45.35 V), and high endurance for voltage‐erasing and light‐programming. These findings demonstrate the high application potential of nanographenes in the field of optoelectronics. In addition, the working principle of these hybrid nanographene‐organic structured heterojunction phototransistor memory devices is described which provides new insight into the design of high‐performance organic phototransistor devices.

## Graphical abstract

Ultrasensitive organic phototransistor memory devices that can be programmed by white light and erased by applying a negative gate voltage are fabricated with a heterojunction structure of azulene‐embedded helical nanographene and 2,9‐didecyldinaphtho[2,3‐*b*:2′,3′‐f]thieno[3,2‐*b*]thiophene (C10‐DNTT). The fabricated phototransistor devices demonstrate high photosensitivity, a large memory window, fast programming speed, a long retention time, and excellent multiple switching stability.

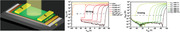

## 1 Introduction

Organic semiconductors possess advantages such as being light‐weight, low‐cost, and mechanically flexible with potential application in wearable electronics.^[^ ^1^ ^]^ The use of organic field effect transistors as organic phototransistors (OPTs) has attracted considerable attention since such devices can be energy‐saving, have ultrafast transmission characteristics, and are promising candidates as elements in next‐generation communication electronics.^[^ ^2^, ^3^, ^4^, ^5^, ^6^ ^]^ Based on the volatility, OPTs can be categorized as photodetectors (sensor memory), artificial synapses (short‐term memory), and memory devices (long‐term memory).^[^ ^7^ ^]^ The latter ones have been considered as the most innovative products of the electronic community since they have a number of advantages such as nonvolatile storage, low‐power consumption, and high reliability.^[^ ^7^ ^]^ For OPT memory devices, the presence of charge trapping sites is desired. Organic electrets, inorganic nanoparticles, and nanostructured defects are the main methods for creating charge traps.^[^ ^7^, ^8^, ^9^, ^10^, ^11^ ^]^ Organic electrets have a number of advantages in their flexibility, the ease of processing, and the variety of new organic materials that can be chosen. Therefore, novel organic photoactive electrets with high photosensitivity and good charge trapping ability are needed to be synthesized and studied to meet the high requirements of the fast‐developing technology.

The trapping ability of traditional ferro‐, piezo‐, and pyroelectric polymeric electrets has been studied in the past.^[^ ^12^, ^13^ ^]^ Recently, new functional polymers and small molecules have been synthesized and applied as electrets in OPT memory devices.^[^ ^4^, ^5^, ^14^, ^15^ ^]^ Nanographenes (NGs) are emerging as promising photoactive and charge trapping materials for electronic and optoelectronic applications, e.g., isolated nanographene was used as the charge trapping nanofloating gate for memory devices,^[^ ^16^ ^]^ and graphene–nanographene heterostructures were applied in photodetector devices.^[^ ^17^ ^]^ As for the trapping properties, the charge trapping ability of NGs is related to their molecular size, shape, and configuration. When NGs work as floating gate, smaller sizes increase its trapping density.^[^ ^16^ ^]^ While, among the different fabrication methods of NGs, such as cutting of graphene sheets, unzipping of carbon nanotubes and chemical vapor deposition, the bottom‐up synthesis method is the most appealing one as it is easier to control the molecular size and side groups additionally to the good reproducibility.^[^ ^18^, ^19^ ^]^ Besides the growing interest in NGs, their application in devices is still in its infancy.

**Figure 1:**
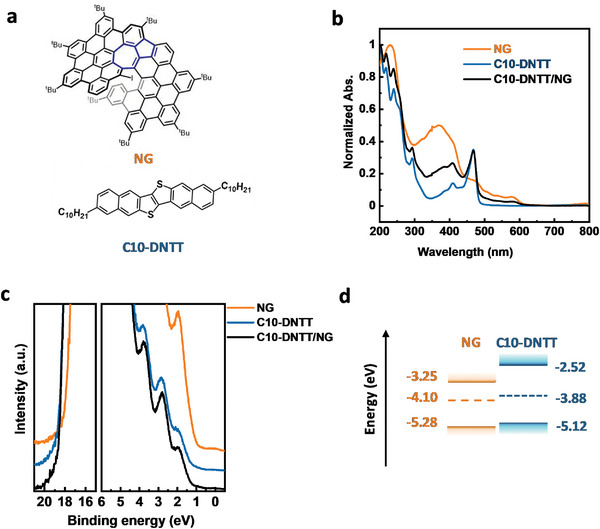
a) Molecular structure of NG and C10‐DNTT. b) UV–vis spectra of NG, C10‐DNTT, and C10‐DNTT/NG heterojunction thin films. c) UPS spectra of NG, C10‐DNTT, and C10‐DNTT/NG thin films. d) Energy level diagram of the NG thin film and the C10‐DNTT thin film.

In this context, we report novel OPT memory devices based on bottom‐up synthesized soluble azulene‐embedded helical nanographene (NG) and the well‐known small molecular semiconductor 2,9‐didecyldinaphtho[2,3‐*b*:2′,3′‐*f*]thieno[3,2‐*b*]thiophene (C10‐DNTT), the chemical structures are shown in **Figure** **1**a. In the device, C10‐DNTT works as the semiconductor, while NG functions as the charge trapping electret. The NG was selected as the electret because of its low bandgap and amorphous layer morphology, which are desired for optical devices and large charge trapping capacity, respectively.^[^ ^20^, ^21^ ^]^ The fabricated Si/SiO_2_/NG/C10‐DNTT/Au bottom‐gate/top‐contact (BGTC) OPT memory devices can be programmed by white light and erased by applying a negative gate voltage. The experimental results demonstrate that NG‐based OPT memory devices exhibit a high light sensitivity, low readout voltage, and nonvolatile memory properties with long retention times.

## 2 Results and Discussion

### 2.1 Characterization of NG and C10‐DNTT Thin Films

Before discussing the NG‐based OPT memory devices in the section below, here we first describe the measured physical properties of the devices’ thin films. Figure 1b shows the ultraviolet–visible (UV–vis) spectra of NG, C10‐DNTT, and C10‐DNTT on NG structured heterojunction (C10‐DNTT/NG) thin films. The absorption band of NG is in the range of 200–603 nm and the calculated optical bandgap is 2.03 eV. C10‐DNTT has a relatively narrow absorption band between 200 and 487 nm with an optical bandgap of 2.6 eV. In addition, the heterojunction film showed the complementary absorption spectrum of the two materials. We also investigated the energy band of NG and C10‐DNTT thin films with ultraviolet photoelectron spectroscopy (UPS) measurements (Figure 1c). The highest occupied molecular orbital (HOMO) and work function of NG are 5.28 and 4.10 eV, while the HOMO and work function of C10‐DNTT are 5.12 and 3.88 eV. According to the UV–vis and UPS results, we drew the energy level diagram of NG and C10‐DNTT thin films, as displayed in Figure 1d. The energy levels of C10‐DNTT and NG are in good agreement with reported values in the literature.^[^ ^22^, ^23^, ^24^ ^]^

**Figure 2:**
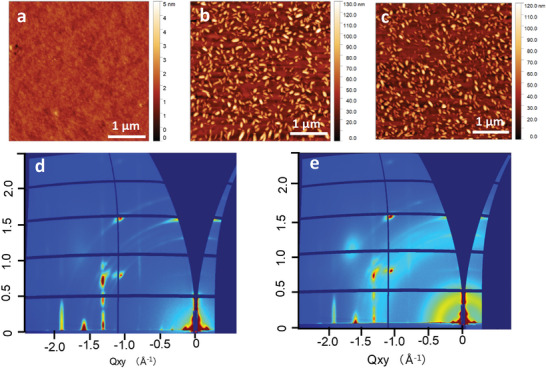
AFM images of a) NG, b) C10‐DNTT, and c) C10‐DNTT/NG. 2D GIWAXS images of d) C10‐DNTT and e) C10‐DNTT/NG.

Atomic force microscope (AFM) and grazing incidence wide‐angle X‐ray scattering (GIWAXS) were employed to investigate the impact of the NG layer on the morphology and crystal structure of C10‐DNTT thin film. The surface of the NG layer is very smooth (**Figure** **2**a) with a root mean square roughness of 0.17 nm. The NG layer produces no discernable GIWAXS signal, indicating an amorphous morphology. The reason for this likely lies in the nonplanar helical structure, which impedes a crystalline close‐packing of the molecules.^[^ ^25^ ^]^ The AFM images of C10‐DNTT and C10‐DNTT/NG (Figure 2b,c) are very similar and show a much rougher morphology that is known for vapor‐deposited C10‐DNTT films^[^ ^26^ ^]^ with small crystals growing vertically on top of a C10‐DNTT base film. The GIWAXS diffraction patterns of pure C10‐DNTT and C10‐DNTT/NG stacked films (Figure 2d,e) show characteristic Bragg rods from the base film, which is highly crystalline and well aligned with the respective substrate (SiO_2_ and NG/SiO_2_, respectively) and ring‐like patterns (“arching”) from less well aligned crystals from the top portion of the C10‐DNTT films—those that are visible as bright features in the AFM images. There are some differences between the two GIWAXS images regarding the degree of the disordered film part, but the highly substrate‐aligned 2D powder pattern (Bragg rods) in the C10‐DNTT/NG image device is virtually identical to that from the pure C10‐DNTT film (in peak positions, intensity ratios, and peak widths). This strongly suggests that the buried device‐relevant interface C10‐DNTT/NG is crystallographically identical to that in the pure C10‐DNTT film.

### 2.2 Transfer Characteristics and Working Principle of NG‐Based OPT Memory Devices

**Figure 3:**
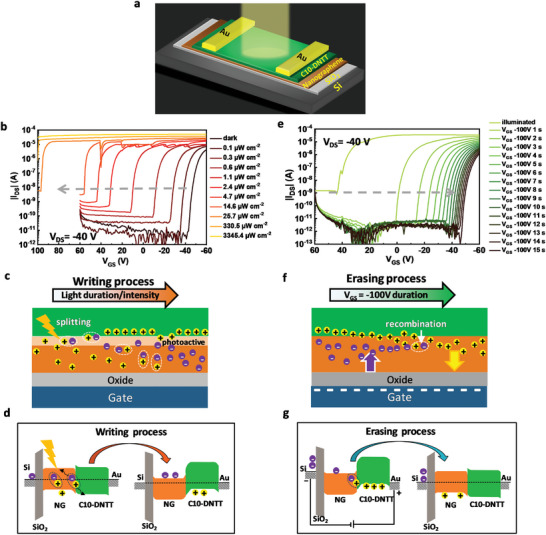
a) Schematic illustration of NG‐based OPT memory devices during measurement. b) Transfer characteristics of the NG‐based OPT memory devices in darkness and under white light illumination with different light intensities. The kinks at ±40 V, ±20 V, and 10^−5^ A were caused by the change in the measurement range of the semiconductor analyzer.^[^ ^33^ ^]^ c) Schematic illustration of the writing process for NG‐based OPT memory devices. d) Diagram of the writing process for NG‐based OPT memory devices. e) Transfer characteristics of the NG‐based OPT memory devices measured after applying ‐100 V for different durations. The “after illumination” curve was measured after exposing the device to 3345.4 µW cm^−2^ white light for 2 min. All measurements were conducted in darkness with a fixed *V*_DS_ of 40 V. f) Schematic illustration of the erasing process for NG‐based OPT memory devices. g) Diagram of the erasing process for NG‐based OPT memory devices.

**Figure** **3**a schematically shows the BGTC architecture of the NG‐based OPT memory devices, which consist of bottom gate SiO_2_ wafer substrates and a C10‐DNTT/NG film stack with gold top electrodes. The detailed fabrication process can be found in the Experimental Section and the thickness of each layer can be seen in Figure S1 (Supporting Information).

The electronic state of the C10‐DNTT/NG film stack devices respond to even just ambient light exposure, and since white light exposure was unavoidable during fabrication, transfer, and contact alignment processes, the as‐fabricated devices were already in a “programmed/written” state. As will be discussed in more detail later, this state can be reverted by an application of a negative gate voltage, erasing the device state. Therefore, in order to assess the impact of light and light power on the device, it was erased by an application of −100 V gate voltage (*V*_GS_) at zero drain voltage (*V*_DS_) for 30 s before recording the light power‐dependent transfer curves in Figure 3b. In the “erased” state, the devices exhibit a fairly significant negative on‐voltage (*V*_on_) of ≈ −43 V and the device was erased before each scan. The transfer curves that were measured under light illumination clearly exhibit a dependence on the white light intensity that the devices are exposed to—by intensity (*P*_inc_) we refer to the light incident power (*P*_opt_) per unit area herein. With increasing light intensity, the transfer curves are more strongly shifted toward positive voltages and their off‐current levels shift to higher values. This readily demonstrates that the NG‐based OPT memory devices are responsive to white light.

The dependence of the device's threshold voltage shift (*∆V*_th_) and off‐current (*I*_off_) on the incident light intensity are plotted in Figure S2 (Supporting Information), with further electrical characteristics being summarized in Table S1 (Supporting Information). A clear gradual increase in *∆V*_th_ and *I*_off_ with increasing light intensity can be seen. According to the empirical equation Δ*V*_th_ = (*nkT*/*q*) × ln[1 + (*nqP*_opt_)/(*I*_dark_ *hv*)] proposed by Muramoto and co‐workers for phototransistor, we fit the obtained *∆V*_th_ dependent on light intensity with equation Δ*V*_th_ = *A* × ln(1 + *B* × *P*_inc_).^[^ ^27^ ^]^ The final obtained equation is Δ*V*_th_ = [27.91 × ln(1 + 4.73 × *P*_inc_)] V, whereas the off current can be described by $I_{\mathrm{off}}=\left[P_{\mathrm{inc}}^{1.17}\;\times \;\left(1.28\times 10^{-10}\right)\right]\;A$—the detailed fitting parameters are shown in Figure S2 (Supporting Information). A direct comparison of the fitting parameters to those reported in other works^[^ ^27^, ^28^, ^29^ ^]^ using this equation is difficult since unlike in those cases, we do not use a single wavelength but rather white light. However, according to the interpretation of the fit,^[^ ^29^ ^]^ the power relationship between the ∆*V*_th_ and *P*_inc_ is due to the strong absorption and high internal quantum efficiency of the photoactive heterojunction—in our case the C10‐DNTT/NG heterojunction.

It is apparent that *V*_th_ shifts to values greater than 100 V if the sample is illuminated by 330.5 µW cm^−2^ light intensity (Figure 3b). The total variation in the threshold voltage (∆*V*_th_) is >140 V. Moreover, the NG‐based OPT memory devices are very sensitive compared with other white light sensitive devices.^[^ ^30^, ^31^ ^]^ *V*_th_ can be changed by 0. 1 µW cm^−2^ light intensity and 0.3 µW cm^−2^ light caused a clear *I*_off_ increase (detailed values can be found in Table S1, Supporting Information). These results indicate that the photoactive layer can efficiently generate charge carriers under low intensity light.

We also fabricated NG‐only and C10‐DNTT‐only BGTC transistor devices. NG‐only devices did not show any conductivity. While Figure S3 of the Supporting Information displays the device architecture of C10‐DNTT‐only devices, the comparison of the performance between C10‐DNTT‐only devices and NG‐based OPT memory is also shown. Under the same light intensity illumination, the *V*_th_ shift in the NG‐based device is much larger than in the C10‐DNTT‐only devices (Figure S3b,e, Supporting Information). A short time exposure to a strong light does not cause a noticeable *V*_on_ shift on the C10‐DNTT‐only transfer curve (Figure S3c, Supporting Information). In addition, it is more difficult to shift *V*_on_ to negative values by applying a −100 V gate voltage in these devices (Figure S3d, Supporting Information). These results indicate that the C10‐DNTT‐only devices would not work well as phototransistor memories and conversely, that the heterojunction between the two materials provides the observed large‐magnitude effects.^[^ ^32^ ^]^ Additionally, compared with C10‐DNTT‐only transistors, the NG‐based OPT memory devices exhibit lower drain current (*I*_DS_), the reason might be both the hole trapping ability of the NG layer^[^ ^4^ ^]^ and the fact that the NG layer, due to its very low mobility, during transistor operation acts as an extra dielectric layer. The capacitances of SiO_2_ and NG/SiO_2_ are displayed in Figure S4 (Supporting Information).

In the following we want to discuss the physical working principle that we find most consistent with the observed device behavior. Figure 3c,d displays the working principle schematically. The key property that enables this principle is that the NG layer can act as a charge trapping layer/electret for both holes and electrons. Before the light illumination, the NG layer is either void of trapped charges (empty) or, if it had been subjected to negative gate voltages in the dark (erasing), some of the injected holes that form the transistor's accumulation layer became trapped in the NG layer with long lifetimes. Upon illumination, electron–hole pairs, i.e., excitons, are generated. Those excitons that are generated within the exciton diffusion length of the NG/C10‐DNTT interface can become split because of the energetic offsets at the interface (see energy band diagram in Figure 1d). (Note that the sketch is drawn with the exciton forming in the NG layer, but the final electrical device state after splitting does not depend in which layer the exciton is formed.) After the splitting, electrons are left in the NG layer due to the large energetic barrier between the LUMO levels, which prevents the electrons from moving to the C10‐DNTT layer, while the photoinduced holes experience a small ≈160 meV driving force/barrier from the HOMO level offset to cross into/remain in the C10‐DNTT layer. In the C10‐DNTT layer, these holes cause an increase in the drain current, whereas the photoinduced electrons are trapped in the NG layer, which results in the positive shift of threshold voltage (*V*_th_). If, depending on the previous device state, trapped holes are already populating the NG layer (after an erasing process/application of large negative gate voltage), the increasing concentration of electrons in the same layer leads to the emptying of the NG layer of charges due to recombination. In any case, the illumination with light will lead to an eventual net storage of long‐lived, trapped electrons in the NG layer. In this work, we refer to the process of storing electrons in the NG layer by light illumination—resulting in a light dose‐dependent positive shift of *V*_th_—as writing.

After studying the impact of light intensity on the devices’ electronic properties, we investigated the impact of negative gate voltage on the NG‐based OPT memory devices (Figure 3e). The shifts in *V*_th_ and *I*_off_ as functions of biasing time (*V*_GS_ = −100 V, *V*_DS_ = 0 V) are shown in Figure S5a (Supporting Information). We note that the resulting *V*_th_ is significantly above 0 V even though the curve was measured in dark, indicating there were still electrons trapped in the NG layer from prior exposure to light. With continued biasing at *V*_GS_ = −100 V, *V*_th_ showed a gradual shift toward negative voltages. This negative shift of *V*_th_ originates from the elimination of trapped electrons that recombine with injected holes in the accumulation layer.^[^ ^34^, ^35^ ^]^ The off current decreased to 10^−11^ A after applying *V*_GS_ = −100 V for 1 s and did not show further change after applying *V*_GS_ for a longer time which suggests that the channel has become completely depleted of mobile holes. The underlying mechanism of this erasing process is illustrated in Figure 3f,g. Previously photoinduced electrons might be trapped in the NG layer before the application of a negative gate voltage. In that case, with the application of a negative bias there is a driving force for these long‐lived trapped electrons to migrate to and concentrate at the interface to C10‐DNTT, which makes a recombination with holes injected into the transistor channel more efficient, resulting in the *V*_th_ shift to 0 V. After elimination of all trapped electrons, a net density of holes becomes trapped in the NG layer, resulting in a *V*_th_ shift further to the negative values.

We also studied the impact of various negative erasing gate biases with the results summarized in Figure S5b (Supporting Information). We found that already a gate bias of −20 V can cause a negative *V*_th_ shift, indicating that even a small/moderate gate bias can initiate the erasing process, i.e., the removal of the trapped electrons from the NG layer.

To explore the impact of the applied gate voltage during the transfer sweeps, measurements with various gate voltage ranges were carried out and the results are displayed in Figure S5c,d (Supporting Information). The *I*_DS_ obtained in the *V*_GS_ range of 40 to −20 V did not show any obvious increase, meaning that the *V*_on_ is ≥20 V. The *V*_on_ of curves measured in the range of 40 to −40 V and 40 to −60 V are −20 V. The *V*_on_ showed a negative shift with further increase of the *V*_GS_ range and finally reached −47 V. We also tested the response of the NG‐based OPT memory devices to a high positive gate bias. As displayed in Figure S6 (Supporting Information), +100 V gate bias did not cause a noticeable *V*_on_ shift, indicating that electrons cannot be directly injected into the NG layer because of the large electron injection barrier.^[^ ^36^ ^]^ Overall, these results indicate that positive gate voltages cannot cause a positive *V*_on_ shift, and that a high negative gate voltage is needed to inject holes into the NG layer.

**Figure 4:**
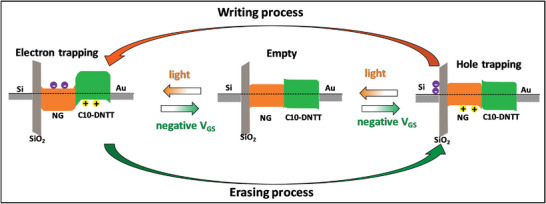
Schematic diagram showing the operation mechanism of NG‐based OPT memory devices.

Based on the above experimental results, we summarize the working principle here to explain how the NG‐based devices work as optical memories, and the working diagram is depicted in **Figure** **4**. If one would for simplicity assume that the devices were to be entirely fabricated in the dark, there would be no electrons or holes trapped in the NG layer. In other words, before exposure to light or application of a gate voltage, the device state is empty. Under illumination, excitons are formed, which upon reaching the NG/C10‐DNTT interface can split into mobile holes in the C10‐DNTT layer and electrons that exhibit very low mobility in the NG layer (electron trapping state). These trapped electrons can be removed by recombination due to the flooding of the channel region with injected holes at a sufficiently large negative gate voltage, returning the device to the empty state or populating the NG layer with trapped holes. Any holes that are trapped in the NG layer after an application of a negative gate bias can then again be eliminated by illuminating the device with light.

### 2.3 Output Characteristics and Evaluation of the Optical Properties

**Figure 5:**
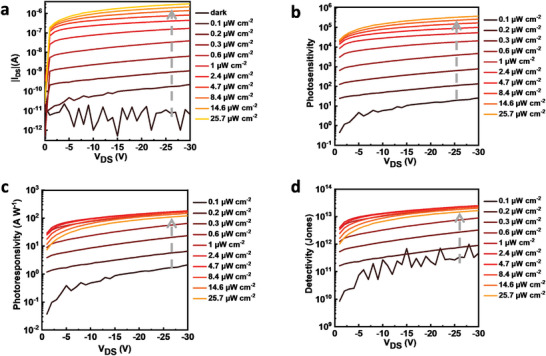
a) *I*_DS_–*V*_DS_ curves of NG‐based OPT memory devices in darkness and under different white light intensities (*V*_GS_ = 0 V). b) Photosensitivity as a function of *V*_DS_. c) Photoresponsivity as a function of *V*_DS_. d) Specific detectivity versus *V*_DS_.

To study the impact of light on the output characteristics of NG‐based OPT memory devices, *I*_DS_(*V*_DS_) measurements were carried out in dark and under different light intensity illuminations at *V*_GS_ = 0 V, as displayed in **Figure** **5**a. The threshold voltage of the device was determined to be −2 V right before the light dependent experiments. In the dark, the measured current was in the range of 10^−12^–10^−11^ A, confirming that the device is in the off‐state. Upon illuminating the device with a white light of the intensity of 0.1 µW cm^−2^, the measured *I*_DS_ increased by one order of magnitude, indicating the device has been successfully written by the weak light. Furthermore, devices exhibit a substantial increase in *I*_DS_ upon increasing the light intensity at the same *V*_GS_ and *V*_DS_. The origin of this behavior is the increasing number of trapped electrons in the NG layer with increasing light intensity, which results in a higher built‐in electric field which, in turn, similar in effect to the external gate electric field effect, increases the source–drain current *I*_DS_.

For OPT devices, in addition to the electrical characteristics, the optical characteristics are also important parameters. It is rather common in the literature to calculate these characteristics based on transfer curves, where in most cases both drain and gate voltage are applied.^[^ ^16^, ^37^, ^38^ ^]^ But for FET structure devices, we think it is better to study the built‐in electric field, which is caused by the trapped electrons/holes, without applying a gate voltage to avoid the impact of the gate‐induced electric field. In addition to that, as mentioned above, a small gate bias leads to the recombination of holes and electrons. Therefore, we investigated the photosensitivity, photoresponsivity, and specific detectivity of NG‐based OPT memory devices with the *I*_DS_–*V*_DS_ curves obtained at *V*_GS_ = 0 V.

The device photosensitivity *P*, which is defined as the signal‐to‐noise ratio, is given by Equation (1)

(1) $$P\;=\frac{\mathrm{signal}}{\mathrm{noise}}\;=\frac{\left(I_{d,\mathrm{ph}}-I_{d,\mathrm{dark}}\right)}{I_{d,\mathrm{dark}}}\;$$

where *I*_d,ph_ and *I*_d,dark_ are *I*_DS_ under illumination and in dark, respectively.^[^ ^31^, ^32^, ^39^, ^40^ ^]^ Figure 5b plots *P* as a function of *V*_DS_. *P* increases with increasing incident light power, reaching a maximum of 3.6 × 10^5^ at *V*_DS_ = −30 V under 25.7 µW cm^−2^ incident light power, indicating an excellent photosensitivity of the NG‐based OPT devices. Despite the high sensitivity, the devices can work at a low readout voltage since clear steps can be seen at *V*_DS_ = −1 V for curves measured under different light intensities.

The photoresponsivity *R* reflects the performance in terms of optical power converted into electrical current and can be calculated from Equation (2)

(2) $$R\;=\frac{I_{\mathrm{ph}}}{P_{\mathrm{opt}}}\;=\frac{\left(I_{d,\mathrm{ph}}-I_{d,\mathrm{dark}}\right)}{P_{\mathrm{inc}}A}\;$$

where *I*_ph_, *P*_opt_, *P*_inc_, and *A* are the photocurrent, power of the incident light, incident light intensity, and area of the active region, respectively. We note that this equation must be used very carefully to calculate the *R* for electric field transistor‐based optical devices since *R* can be very high as long as the *I*_d,ph_ is high even if the light only causes a small current increase.^[^ ^7^, ^41^, ^42^, ^43^ ^]^ The *I*_DS_–*V*_DS_ curves at a fixed *V*_GS_ of 0 V have been utilized here to exclude the exaggeration of *R*, which can be caused by the gate‐induced electric field. The relationship of *R* and *V*_DS_ under different light intensities is plotted in Figure 5c. The photoresponsivity increased with the increasing *V*_DS_ and peaked at 1 µW cm^−2^ light intensity, with the highest *R* being 160 A W^−1^. The diminishing photoresponsivity at higher light intensity could be attributed to two mechanisms: the saturation of the charge storage capacity of the NG layer, and the density‐dependent electron–hole recombination, which is higher at larger number of photoinduced charges.^[^ ^40^, ^44^ ^]^

The specific detectivity *D* ^*^ is calculated from Equation (3)

(3) $$D^{*}=\frac{\left(R\sqrt{A}\right)}{\sqrt{(2qI_{d,\mathrm{dark})}}}\;$$

where *q* is the electron charge and with the assumption that the total background noise is dominated by the shot noise from the dark current.^[^ ^41^ ^]^ We plot the D* versus *V*_DS_ curves in Figure 5d. It can be seen from Equation (3) that D* is related to the photoresponsivity (*R*), thus exhibiting the same trend with respect to the light intensity. The highest obtained D* is 2 × 10^13^ Jones.

The photo characteristics of the NG‐based OPT memory devices that calculated from the *I*_DS_–*V*_DS_ curves with *V*_GS_ = 0 V are competitive compared to reports from literature which are usually calculated from higher gate–source and drain–source voltages.^[^ ^7^, ^31^, ^43^ ^]^ These results demonstrate that the NG‐based OPT memory devices are excellent optical devices that can work at low voltage and are responsive to ultraweak white light.

### 2.4 Memory Properties of NG‐Based OPT Memory Devices

Besides the “vertical” memory window—the spread of the current signal between the different device states—the hysteresis window is defined as the change in threshold voltage between the forward and the backward sweeps caused by the net charge carrier trapping/detrapping is another important parameter for transistor memory devices.^[^ ^8^ ^]^ Hence, we measured the transfer curves in forward and backward directions (dual‐sweep) of the device under different light intensities and after exposure to different light doses.

The dual‐sweep curves of NG‐based OPT memory devices in dark and under different light exposures are shown in Figure S7a of the Supporting Information, the hysteresis window and the ∆*V*_th_ versus light intensity are plotted in Figure S7b of the Supporting Information. The highest hysteresis of 25 V is achieved when the light intensity was 0.6 µW cm^−2^. For even higher light intensities, the backward curves are not typical transfer‐characteristic anymore and the device cannot be switched off by reducing the gate voltage, indicating a high built‐in electrical field from a high density of trapped, photoinduced electrons in the NG layer. The results indicate that for the phototransistors one can think of the effective gate electric field as seen by the channel region as being caused by two sets of charges: both $Q_{\mathrm{gate}}^{e}$, the external gate electrons, and the internal photoinduced electrons ($Q_{\mathrm{photo}}^{e}$). Under low light intensities, the effective gate electric field is then dominated by $Q_{\mathrm{gate}}^{e}$ and the transfer curves are controlled by the external gate bias (*V*_GS_), resulting in conventional transistor transfer curves. On the other hand, under significant light illumination, the $Q_{\mathrm{photo}}^{e}$ charges cause a high internal contribution to the effective gate electric field so that a high *I*_DS_ current is obtained even at low or positive external gate bias *V*_GS_.

**Figure 6:**
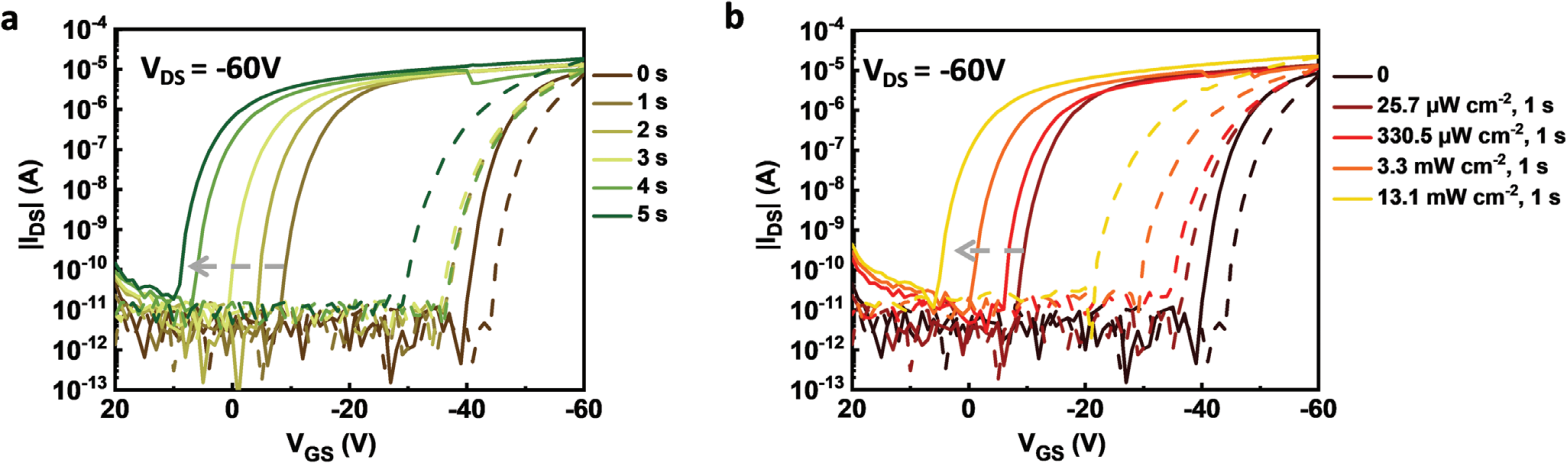
Transfer characteristics, measured in darkness for all curves. Solid lines are forward sweeps and dashed lines are reverse sweeps. a) Forward and reverse sweeps of NG‐based OPT memory devices after exposure to 25.7 µW cm^−2^ for different durations. b) Forward and reverse sweeps of NG‐based OPT memory devices after premeasurement exposures to different light intensities for 1 s.

The dual‐sweep curves after a brief exposure to light were studied to investigate the impact of light exposure history on NG‐based OPT memory devices. **Figure** **6**a displays the transfer curves measured in dark after different exposure durations to a light intensity of 25.7 µW cm^−2^, revealing a gradual shift of *V*_th_ in the direction of positive voltages with increasing exposure times. A variation of 49.75 V was achieved after 5 s light exposure, and the highest hysteresis was 43.29 V, detailed data are summarized in Table S2 (Supporting Information). Moreover, the transfer curves after exposure to different light intensities for 1 s are shown in Figure 6b, ∆*V*_th_ increased with the increase of light intensity, other detailed electrical characteristics can be found in Table S3 (Supporting Information).

The large memory window and hysteresis of the NG‐based phototransistor are attributed to the high photosensitivity and strong electron/hole trapping capability of the NG layer. In addition, the results also indicate that NG‐based OPT memory devices are not only sensitive to light but also can “remember” light exposure history and light dose, indicating NG‐based OPT memory devices are promising candidates for photodosimeter application.

**Figure 7:**
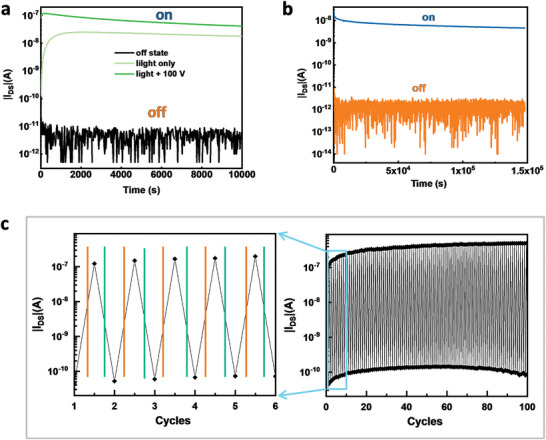
All *I*_DS_ values were measured in dark with *V*_GS_ = 0 V, *V*_DS_ = −3 V. a) Retention time test of NG‐based OPT memory devices, programmed with 330.5 µW cm^−2^ for 1 s, programmed by sequential light (330.5 µW cm^−2^ for 1 s) and positive gate voltage (+100 V gate bias for 1 s), and −100 V gate bias for 1 s for erasing. Measurements were carried out in ambient conditions. b) Retention time characteristics of NG‐based OPT memory devices programmed with ambient light (566.5 µW cm^−2^) for 2 s and off state (−100 V 1 s). c) WRER cycles of NG‐based OPT memory devices. Left: enlargement of the first 5 cycles (the green line represents −50 V gate bias for 1 s; the orange line represents exposing the device under 3345 µW cm^−2^ light for 1 s). Right: 100 WRER cycles.

For practical applications of nonvolatile memory devices, retention time, and endurance/reliability are important parameters, which indicate long‐term memory stability and multiple switching stability, respectively.^[^ ^2^, ^4^, ^5^, ^12^, ^45^, ^46^ ^]^ The evaluation of these memory properties of NG‐based OPTs memory devices is shown in **Figure** **7**. For these measurements, *V*_th_ < 0 V is defined as “0” (off) state, and *V*_th_ > 0 V is defined as “1” (on) state.

Figure 7a,b plots the ability of the NG layer to retain data with the retention time after the writing and erasing operations. Two “on state” readout curves are shown in Figure 7a: one where the device is getting written/programmed by light exposure only and one where the device is written by both light exposure and a subsequent positive gate voltage. A slower rise in current upon time for the first 500 s can be seen for the light‐only programmed curve The reason for this is that the hole/electron generation rate is higher than their recombination rate, and the total number of photoinduced electrons/holes increases upon time. Subsequently, a stable stage can be seen when recombination rate equals generation rate, followed by a rather slow drop‐off over time. A higher memory on/off ratio is obtained when the device is programmed with light and +100 V gate voltage and the current increase time is then imperceptible. That is because the photoinduced electrons moved drift further away from junction into deeper located trapping sites, driven by the electrostatic force from the positive gate voltage, and as a result, the recombination rate and the generation rate reach a balance within a shorter time frame. Figure 7b displays the NG‐based OPT memory devices when programmed without using a dedicated light source but simply by ambient light in order to demonstrate that the programming of the OPT memory devices is not limited to white LED light sources. From the retention tests we see that the memory on/off ratio is above 1.0 × 10^3^ even though the readout voltage is very low (*V*_GS_ = 0 V and *V*_DS_ = −3 V) and remains largely the same after 1.5 × 10^5^ s (in Figure 7b), demonstrating the excellent data durability of the NG‐based OPT memory devices. Additionally, the devices were periodically exposed to a writing white light of 3345 µW cm^−2^ for 1 s and with an erasing gate voltage of −50 V for 1 s to test the multiple write–read–erase–read (WRER) stability. No obvious decay was observed after the dynamic on/off switching for 100 cycles (Figure 7c), suggesting that the NG‐based OPT memory devices possess a good stability.

We compared the performance of NG based OPT memory devices with previously reported high‐performance organic phototransistor memory devices (**Table** **1**).^[^ ^6^, ^36^, ^47^, ^48^, ^49^, ^50^, ^51^, ^52^, ^53^, ^54^ ^]^ Compared to the reported devices, the NG‐based OPT memory devices are more sensitive than other reported devices. For both modes of illumination—constant exposure and short‐time exposure—the NG‐based OPT devices produce a significant response, even at low incidence light intensity. The resulting incident light power normalized memory window (∆*V*_th_/P) is much larger than that of other reported organic phototransistor devices. In addition, the retention time is outstanding compared with reported organic phototransistor memories.

**Table 1:** The performance of different organic phototransistor memory devices

Channel	Programming	Erasing	Memory window ∆*V*_th_ [V]	Light intensity	Light time	∆*V*_th_/P [V cm^2^ mW^−1^]	Retention time [s]	Refs.
DNTT	Light + bias	Bias	170	10 mW cm^−2^	Continuously	17	5 × 10^4^	[36]
C60/PTCDA	Light	Bias	69	0.13 mW cm^−2^	Continuously	530.7	N/A	[47]
Pentacene	Light	Bias	82	25 mW cm^−2^	Continuously	3.28	10^4^	[6]
C10‐DNTT	Light	Bias	140	0.025 mW cm^−2^	Continuously	2600	1.5 × 10^5^	This work

Channel	Programming	Erasing	Memory window [V]	Light intensity	Light time	∆*V*_th_/P/T [V cm^2^ mW^−1^ s^−1^]	Retention time [s]	Refs.
IDTBT	Light	Bias	57.7	N/A	1 s	N/A	10^4^	[48]
C12‐BTBT	Light + bias	Bias	80	N/A	N/A	N/A	10^4^	[49]
Pentacene	Light	Bias	80	25 mW cm^−2^	1 s	3.2	4 × 10^3^	[50]
BBTNDT	Light	Bias	60	40 mW cm^−2^	10 s	0.15	2 × 10^4^	[51]
WG_3_/pentacene	Light	Bias	45	5 mW cm^−2^	1 s	9	10^4^	[52]
Pentacene	Light	Bias	50	12.3 mW cm^−2^	30 s	0.14	10^4^	[53]
PDVT‐8	Light	Bias	42	0.5 mW cm^−2^	0.5	168	2 × 10^5^	[54]
C10‐DNTT	Light	Bias	35	0.025 mW cm^−2^	1 s	1400	1.5 × 10^5^	This work
			45	13.1 mW cm^−2^	1 s	3.42		

Note: The comparison of more studies can be found in a recent published review.^[^ ^7^ ^]^

All of these results indicating such hybrid heterojunctions are promising candidates for write‐once‐read‐many (WORM) nonvolatile photomemories.

## 3 Conclusion

In summary, high‐performance NG‐based OPT memory devices that can be programmed by white light were developed with high photosensitivity (3.6 × 10^5^), a large memory window exceeding 140 V under light, fast programming speed (1 s), a long retention time (>1.5 × 10^5^ s) and good multiple switching stability (>100 demonstrated). We also found that the presence of the heterojunction interface is essential for the photoresponsivity of the fabricated devices. We proposed a working principle based on the experimental results of this work and former studies, which can be helpful for designing and fabricating high‐performance phototransistor devices. Additionally, the electrical characteristics of the devices change with the incident light dose, indicating the promising application of such devices as photo‐dosimeters and multiple states data storage devices.

## 4 Experimental Section

### Materials

NG was synthesized as reported,^[^ ^24^ ^]^ C10‐DNTT was purchased from Lumtec and used without further purification.

### Characterization of Thin Films and Devices

AFM images were obtained in tapping mode with a Flex‐Axiom from Nanosurf. Thickness was measured with a profilometer (DektakXT, Bruker). GIWAXS measurements of C10‐DNTT/NG and C10‐DNTT were carried out at SIRIUIS beamline at SOLEIL, France. The sample‐to‐detector distance was 306 mm and an X‐ray beam energy of 10 keV was used. The incident angle was 0.14°. All images were recorded using a Pilatus 1M detector. The NG GIWAXS measurements were done at NCD‐SWEET beamline at ALBA, Spain, with a sample‐to‐detector distance of 180 mm and an X‐ray energy of 12.4 keV. The incident angle was set as 0.12° and the images were recorded using a Rayonix (LX255‐HS) detector. UPS was performed with a Thermofisher Escalab 250Xi system, a He^I^ lamp (26.3 eV), −5 V bias, and a passing energy of 2 eV were applied for measuring. The work function (*E*_Ψ_) was calculated by subtracting the cutoff values of the secondary photoemission onset from the photon energy of 26.3 eV, with the formula

(4) $$E_{\Psi }=\;26.3-E_{\mathrm{cutoff}}$$

Valence band minimum (VBM) onset value was extracted from the UPS spectra by extrapolating the slope of the photoemission onset and reading the value from the intersection with the *x*‐axis. Then, the VBM value was calculated with the formula

(5) $$E_{\mathrm{VBM}}=E_{\Psi }\;+E_{\mathrm{VBM},\mathrm{onset}}$$

Si was used as the substrate for above mentioned measurements.

UV–vis spectra were obtained by using a spectrophotometer (Cary 5000, Agilent) and quartz slides were used as the substrate. The optical bandgap was evaluated from the absorption spectrum using the following Tauc relation

(6) $${\left(\alpha hv\right)}^{2}=\;C\left(hv-E_{G}\right)$$

where *C* is a constant, *C* = 1 for solid samples, *α* is the absorption coefficient, *h* is the Planck's constant, *v* is the photon frequency, *E*_G_ is the optical bandgap and obtained by extrapolating the straight portion of the curves to zero absorption coefficient value.

### Device Fabrication

NG in CHCl_3_ (8 mg mL^−1^) was shear coated (5000 µm s^−1^) at room temperature onto highly n‐doped silicon wafers with a SiO_2_ (300 nm) layer under ambient conditions. The SiO_2_/Si substrates were successively cleaned with acetone and ethanol for 10 min, and then dried with nitrogen. A C10‐DNTT (25 nm, 0.025 nm s^−1^) film was thermally evaporated on top of the NG layer, and Au (50 nm, 0.15 nm s^−1^) was evaporated as the top source and drain contacts.

### Characterization of NG‐Based OPT Memory Devices

The retention measurements programmed by room light were performed with a Keysight B1500 semiconductor analyzer, and the ambient light intensity was 566.5 µW cm^−2^. The quantified photoresponse measurements were conducted with a HP 4145 semiconductor analyzer controlled by SweepMe!. White light illumination was performed using an LED (MWWHLP1 – 3000 K, Thorlabs) with an emission spectrum from 400 to 800 nm, and light intensity was measured with a Si reference diode (SRC‐1000‐RTD‐QZ, VLSI Standards). *V*_on_ is the gate voltage where *I*_DS_ enters the regime of weak accumulation in the transfer curves. The threshold voltage (*V*_th_) was obtained from the intersection of the linear fit of the *I*_DS_^1/2^ curve and the *x*‐axis, *I*_off_ was the average current before *V*_on_. The capacitance was measured with an LCR meter (Keysight E4980).

## Conflict of Interest

The authors declare no conflict of interest.

## Supporting information

Supporting Information

## Data Availability

The data that support the findings of this study are available from the corresponding author upon reasonable request.
