# Supplementary material for: Nanographene‐Based Heterojunctions for High‐Performance Organic Phototransistor Memory Devices
Source: Adv Sci (Weinh). 2023 Mar 30;10(15):2300057. doi: 10.1002/advs.202300057 (PMC10214218; doi:10.1002/advs.202300057)
Supplement: Supplementary file 1 — Supporting Information [file ADVS-10-2300057-s001.pdf]

ADVANCED SCIENCE
Open Access

Supporting Information

for *Adv. Sci.*, DOI 10.1002/advs.202300057

Nanographene-Based Heterojunctions for High-Performance Organic Phototransistor Memory Devices

*Shaoling Bai, Lin Yang, Katherina Haase, Jakob Wolansky, Zongbao Zhang, Hsin Tseng, Felix Talnack, Joshua Kress, Jonathan Perez Andrade, Johannes Benduhn, Ji Ma, Xinliang Feng, Mike Hambsch and Stefan C. B. Mannsfeld**

Supporting Information

Nanographene-based heterojunctions for high-performance organic phototransistor memories

*Shaoling Bai, Lin Yang, Katherina Haase, Jakob Wolansky, Zongbao Zhang, Hsin Tseng, Felix Talnack, Joshua Kress, Jonathan Perez Andrade, Johannes Benduhn, Ji Ma, Xinliang Feng, Mike Hambsch, Stefan C. B. Mannsfeld**

S. Bai, Dr. K. Haase, F. Talnack, Dr. M. Hambsch, and Prof. S. C. B. Mannsfeld
Center for Advancing Electronics Dresden (cfaed), Technische Universität Dresden, Helmholtzstraße 18, 01062 Dresden, Germany
Faculty of Electrical and Computer Engineering, Technische Universität Dresden, Helmholtzstraße 18, 01062 Dresden, Germany.
E-mail: stefan.mannsfeld@tu-dresden.de

L. Yang, Dr. J. Ma, Prof. X. Feng
Center for Advancing Electronics Dresden (cfaed), Technische Universität Dresden, Helmholtzstraße 18, 01062 Dresden, Germany
Faculty of Chemistry and Food Chemistry, Technische Universität Dresden, Helmholtzstraße 18, 01062 Dresden, Germany

Dr. J. Ma, Prof. X. Feng
Max Planck Institute of Microstructure Physics, Weinberg 2, 06120 Halle, Germany

J. Wolansky, Z. Zhang, H. Tseng, J. Kress, and Dr. J. Benduhn
Dresden Integrated Center for Applied Physics and Photonic Materials (IAPP) and Institute for Applied Physics, Technische Universität Dresden, Nöthnitzer Str. 61, 01187 Dresden, Germany

J. P. Andrade

Leibniz Institute for Solid State and Materials Research, Helmholtzstraße 20, 01069 Dresden, Germany

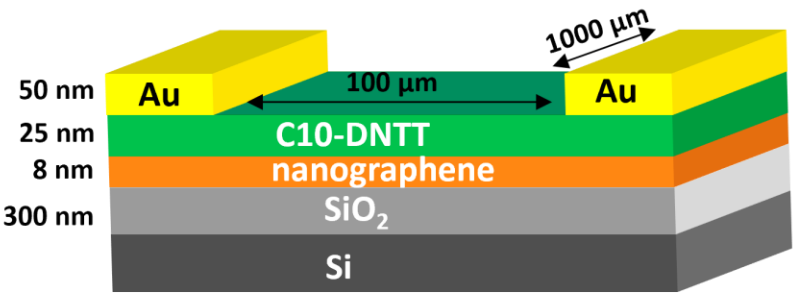

Figure S1. Device structure of the NG-based phototransistor.

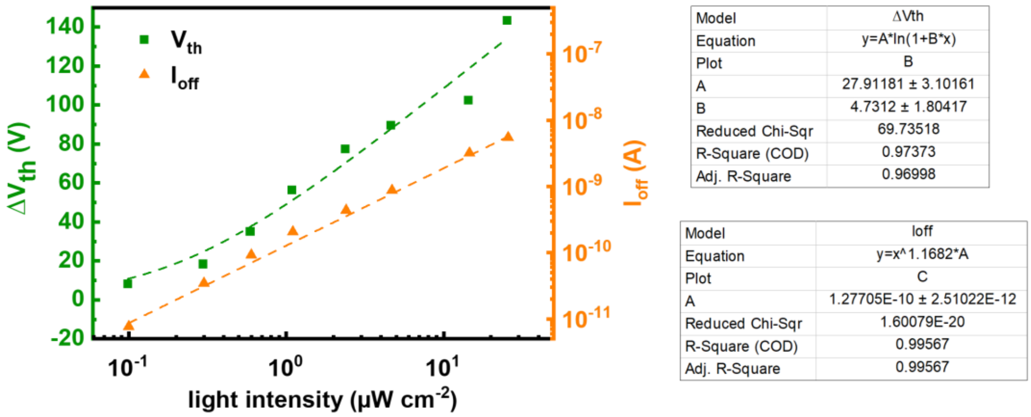

Figure S2. V_{th} and I_{off} versus light intensity. The information of curves measured under high light intensity illuminations (330.5 μW cm^{-2} and 3345.4 μW cm^{-2}) is not shown here because 100 V is the limitation of the measurement setup. Dashed lines are fitting lines.

Table S1. Summary of NG-based OPT memory electrical characteristics under different light intensities.

Light intensity (μW cm^{-2})	V_{on} (V)	V_{th} (V)	ΔV_{th} (V)	I_{off} (A)
0	-42.6	-46.28	0	$9.20*10^{-12}$
0.1	-35.04	-38.03	8.25	$7.70*10^{-12}$

0.3	-25.37	-27.93	18.35	$3.50*10^{-11}$
0.6	-9.65	-11.21	35.07	$9.20*10^{-11}$
1.1	11.53	10.21	56.49	$2.10*10^{-10}$
2.4	31.76	31.28	77.56	$4.40*10^{-10}$
4.7	43.86	43.35	89.63	$8.70*10^{-10}$
14.6	55.92	56.14	102.42	$3.20*10^{-9}$
25.7	97.39	97.15	143.43	$5.50*10^{-9}$
330.5	-	-	-	-
3345.4	-	-	-	-

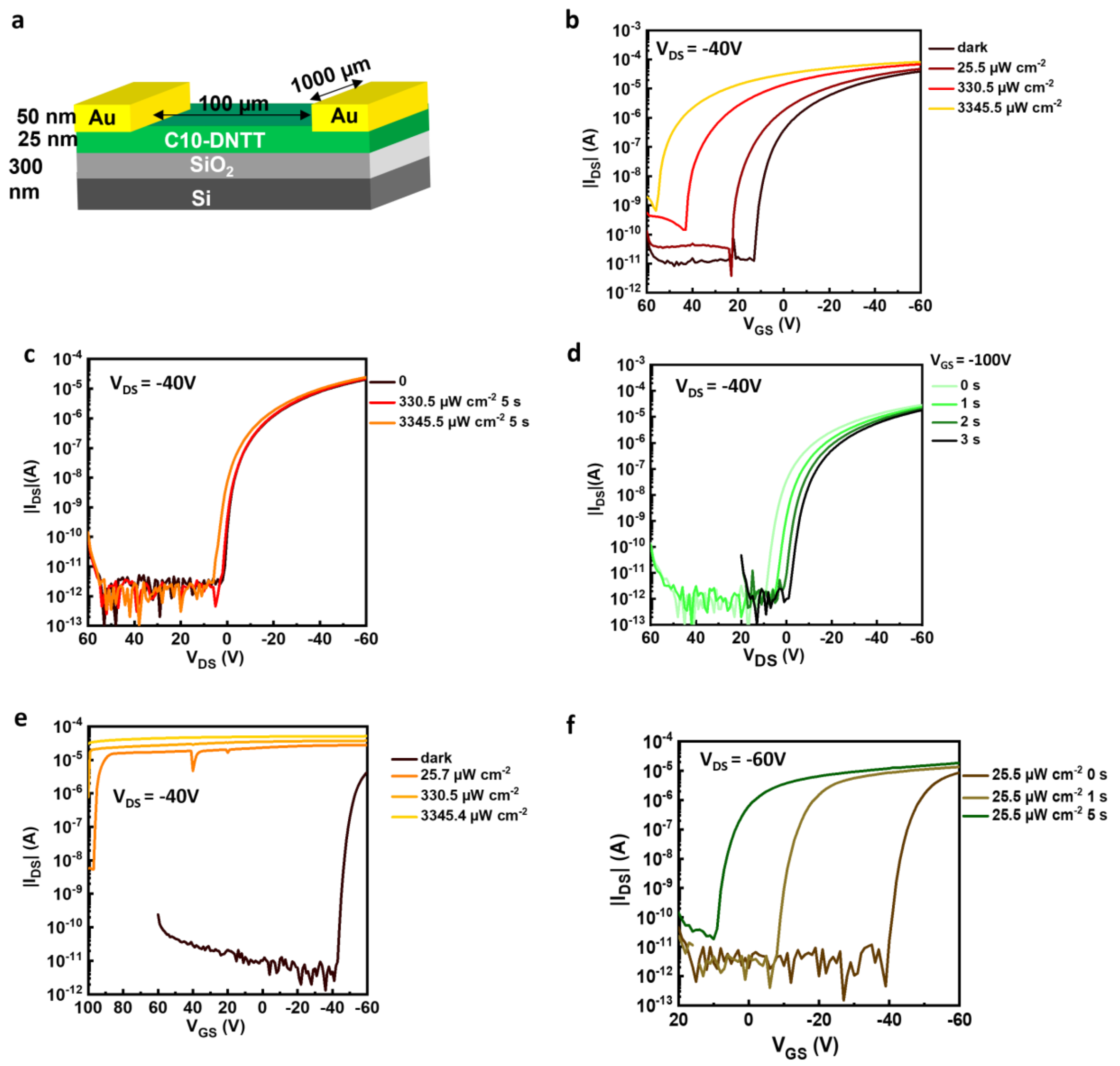

Figure S3. Comparison of C10-DNTT-only device and NG-based OPT memories. (a) Device structure of C10-DNTT-only OFET. (b) Transfer characteristics of a C10-DNTT-only device measured in dark and under white light exposure. (c) Transfer curves of C10-DNTT-only device after 5 s exposure to high intensity of light illumination. (d) The impact of -100 V gate voltage on transfer characteristics of a C10-DNTT-only device. (e) Transfer curves of NG-based OPT memory measure under different light intensity exposure. (f) Transfer curves of NG-based OPT memory devices after exposure to 25.7 µW cm^{-2} for different durations.

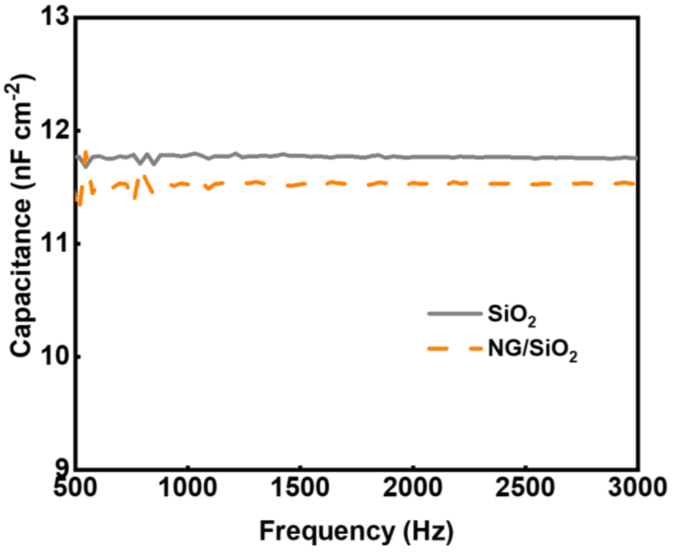

Figure S4. Capacitance versus frequency curves of SiO_2 and NG/SiO_2.

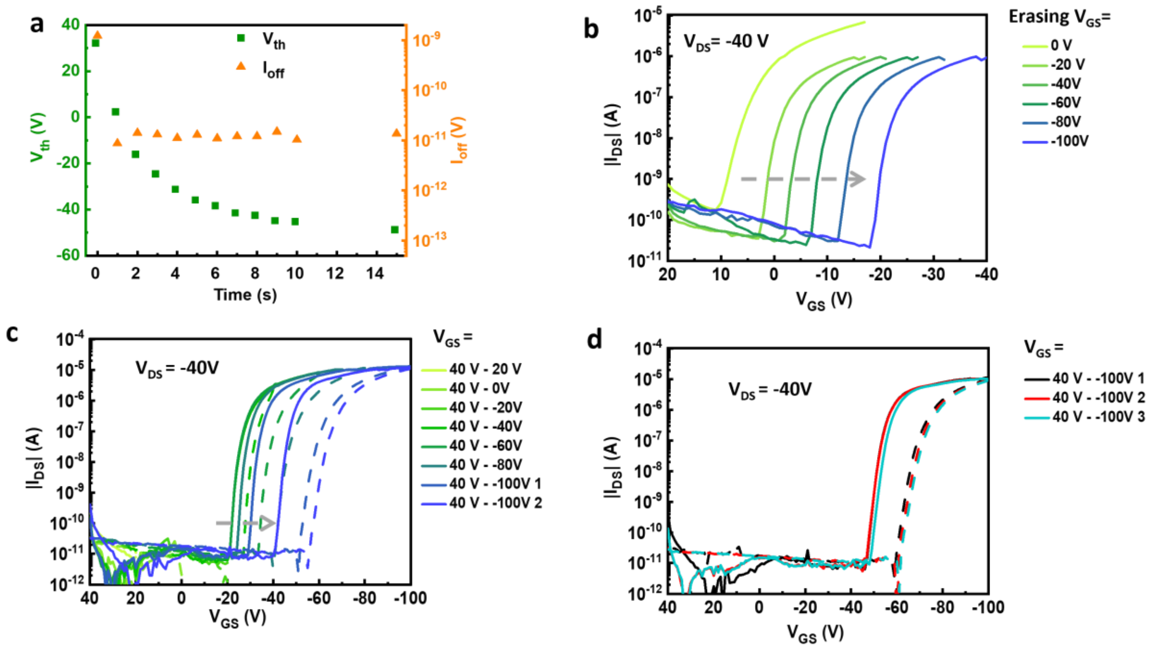

Figure S5. All transfer curves were measured in dark. (a) V_{th} and I_{off} as functions of V_{GS} = -100 V biasing time. (b) Impact of various negative gate voltage on transfer characteristics, applied for 1s before each measurement, and the 0 V curve was measured after ambient light exposure, and, the light intensity of the ambient light was 566.5 μW cm^{-2}. (c) Transfer curves measured in different V_{GS} ranges, measurements were carried out from small range to big range, solid lines are forward sweeps and dashed lines are backward sweeps. The V_{on} was -20 V before measuring. (d) Transfer curves of the same device measured three times, solid lines are forward sweeps and dashed lines are backward sweeps.

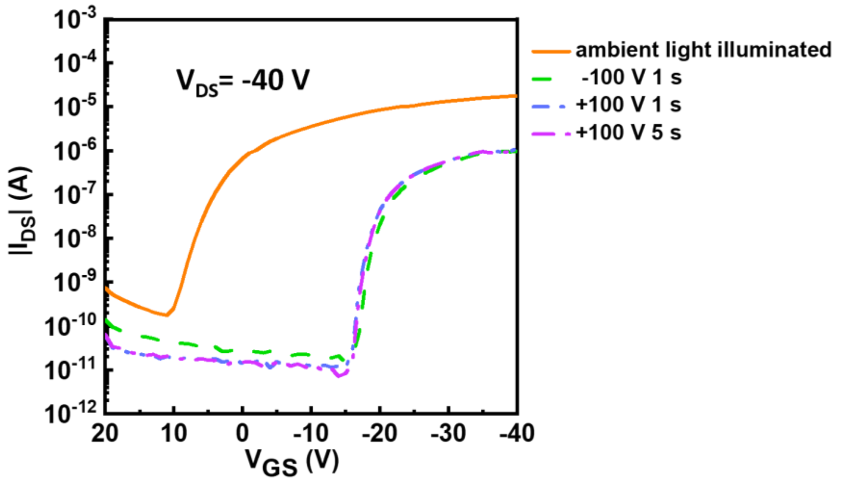

Figure S6. Impact of +100 V gate bias on transfer characteristics of NG-based OPT memory devices.

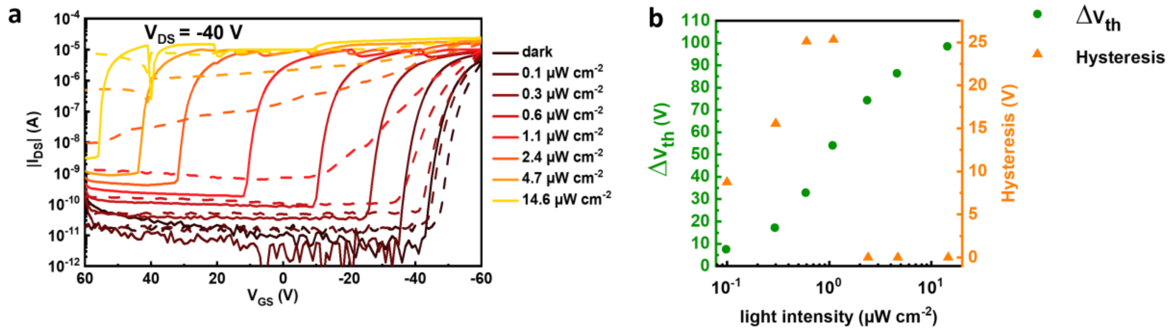

Figure S7. (a) Forward and backward scans of the NG-based OPT memory devices in darkness and under different white light intensities, solid lines are forward sweeps and dashed lines are backward sweeps. (b) ΔV_{th} and hysteresis widow versus light intensity.

Table S2. Summary of NG-base OPT memory devices characteristics after exposure to 25.7 μW cm^{-2} light intensity for different times.

Light intensity (μW cm^{-2})	Time (s)	$V_{on, forward}$ (V)	$V_{th, forward}$ (V)	ΔV_{th} [a)] (V)	$V_{th, backward}$ (V)	Hysteresis window (V)
25.7	0	-39.36	-42.37	0	-48.59	6.22
	1	-7.82	-7.82	34.55	-40.98	33.16
	2	-4.18	-6.22	36.15	-40.97	34.75
	3	0.94	-1.71	40.66	-40.73	39.02

	4	7.09	4.47	46.84	-40.88	45.35
	5	9.88	7.38	49.75	-33.9	41.28

a) memory window calculated with forward curves

Table S3. Summary of NG-based OPT memory characteristics after exposure to different light intensity for 1 s.

Light intensity (μW cm^{-2})	Time (s)	$V_{on, forward}$ (V)	$V_{th, forward}$ (V)	ΔV_{th} [a] (V)	$V_{th, backward}$ (V)	Hysteresis window (V)
0		-39.36	-42.37	0	-48.59	6.22
25.7		-7.82	-11.03	31.34	-40.98	29.95
330.5	1	-6.12	-8.12	34.25	-39.62	31.5
3300		0.06	-2.63	39.74	-34.11	31.48
13100		5.66	3.15	45.52	-25.08	28.23

a) memory window calculated with forward curves
